# Positional Nystagmus after Acute Vertiginous Attack in Meniere’s Disease

**DOI:** 10.3390/audiolres11010007

**Published:** 2021-02-06

**Authors:** Haemin Noh, Dong-Han Lee, Jung Eun Shin, Chang-Hee Kim

**Affiliations:** Department of Otorhinolaryngology-Head and Neck Surgery, Research Institute of Medical Science, Konkuk University School of Medicine, Konkuk University Medical Center, Seoul 05029, Korea; 20170092@kuh.ac.kr (H.N.); 20200189@kuh.ac.kr (D.-H.L.); 20050055@kuh.ac.kr (J.E.S.)

**Keywords:** Meniere’s disease, nystagmus, vertigo

## Abstract

There have been no reports regarding nystagmus observed immediately after the end of an acute vertiginous attack in patients with Meniere’s disease. The aim of this study was to demonstrate positional direction-changing nystagmus in patients with Meniere’s disease, and to discuss the mechanism that underlies this nystagmus. Video-nystagmography was recorded in two patients with definite Meniere’s disease, who showed positional direction-changing nystagmus during the period immediately after a vertigo attack. In one patient, video-nystagmographic recording was conducted 5 h after an episode of vertigo attack, and it showed very weak, persistent positional geotropic direction-changing nystagmus. In the other patient, video-nystagmographic recording was conducted 23 h after an episode of vertigo attack, and it showed very weak, persistent positional apogeotropic direction-changing nystagmus. Our patients exhibited very weak, persistent positional direction-changing nystagmus, which was geotropic in one and apogeotropic in the other. This type of positional nystagmus has been reported in other inner ear disorders and it cannot be clearly explained by typical benign paroxysmal positional vertigo. The change in chemical composition and/or electrolyte concentration of the inner ear fluid, although still unclear, may underlie the production of this characteristic nystagmus in these patients.

## 1. Introduction

In 1861, Prosper Meniere first noted that Meniere’s disease, which is a clinical syndrome that consists of vertigo, loss of balance, and hearing loss, is caused by a lesion of the inner ear. Definite Meniere’s disease is a clinical condition defined by two or more spontaneous vertigo attacks lasting 20 min to 12 h, with audiometrically documented low- to mid-frequency sensorineural hearing loss in the affected ear before, during or after an episode of vertigo [1,2]. In tertiary referral centers, clinicians often see patients in the interictal period, because the vertigo in Meniere’s disease generally lasts less than 12 h. Nevertheless, there have been some reports demonstrating nystagmus during acute vertiginous attacks in Meniere’s disease [3,4,5,6,7]. It is generally accepted that spontaneous nystagmus beats toward the affected ear (‘irritative’ nystagmus) during the early phase and then away from it (‘paralytic’ nystagmus) during the later phase of vertigo attack, although some discrepancies exist regarding the direction of nystagmus during attacks. However, there have been no reports showing nystagmus findings only several hours after the end of acute vertiginous attack in patients with Meniere’s disease. In this study, we present two patients with unilateral definite Meniere’s disease, in whom nystagmus was recorded several hours after the end of an acute vertiginous attack, with a discussion on the characteristics of nystagmus.

## 2. Case Presentation

### 2.1. Case 1

**Figure 1:**
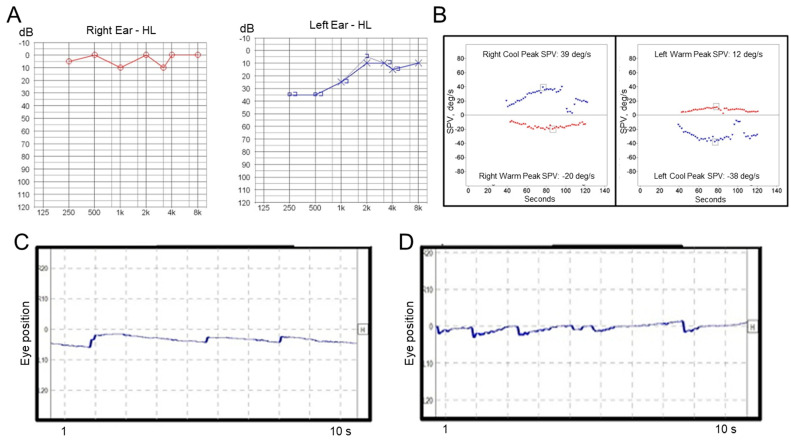
Results of laboratory tests in Case 1. (**A**) Pure tone audiometry shows low-frequency hearing loss on the left side. (**B**) A bithermal caloric test reveals canal paresis of 8% on the left side. Video-nystagmography demonstrates very weak right-beating nystagmus in the right head-roll position (**C**), and left-beating nystagmus in the left head-roll position (**D**).

A 52-year-old man with diabetes mellitus first visited our clinic with a complaint of acute vertigo and aural fullness on the left side. The patient reported that he had experienced vertiginous attacks several times before. He did not complain of headache and denied a past medical history of migraine. An otoscopic examination revealed a normal tympanic membrane, and neurological examination revealed no focal neurologic deficit. Video-nystagmography, which was performed one day after the vertigo subsided, demonstrated weak right-beating spontaneous nystagmus (slow-phase velocity, SPV = 2°/s) without direction change on the positioning maneuvers. Pure tone audiometry showed mild low-frequency hearing loss on the left side (Figure 1A), and the bithermal caloric test revealed no canal paresis on either side (Figure 1B). The patient was treated with systemic steroids and intratympanic steroid injections during the acute stage, and then maintained with betahistine and lifestyle modification. However, after this, the patient experienced repeated vertiginous attacks lasting about 2 h each. He visited our outpatient clinic again with a complaint of acute vertigo, which had started 8 h prior and lasted for about 3 h, with an aggravation of hearing loss and tinnitus on the left side. Examination using video Frenzel goggles, which was performed 5 h after the vertigo subsided, demonstrated persistent geotropic positional direction-changing nystagmus during a head-roll test (Supplementary Video S1). Video nystagmography, which was performed one day after the vertigo subsided, showed very weak, persistent geotropic positional direction-changing right-beating nystagmus (SPV = 1°/s) in the right head-roll position (Figure 1C) and left-beating nystagmus (SPV = 2°/s) in the left head-roll position (Figure 1D). The presence of pseudo-spontaneous and positional nystagmus during head movement in the pitch plane was also investigated [8,9]. The patient exhibited weakly right-beating (SPV = 1°/s) spontaneous nystagmus in a sitting position, and weakly right-beating nystagmus (SPV = 1°/s) in a bowing position and weakly left-beating nystagmus (SPV = 2°/s) in a leaning position. A bithermal caloric test revealed canal paresis of 49% on the left side. A video head impulse test revealed normal values in all the semicircular canals on both sides. The patient was treated with systemic steroids and intratympanic steroid injections. After steroid treatment, the patient did not experience an acute vertiginous attack for 22 months following the last attack, although sensorineural hearing loss on the left side did subside.

### 2.2. Case 2

**Figure 2:**
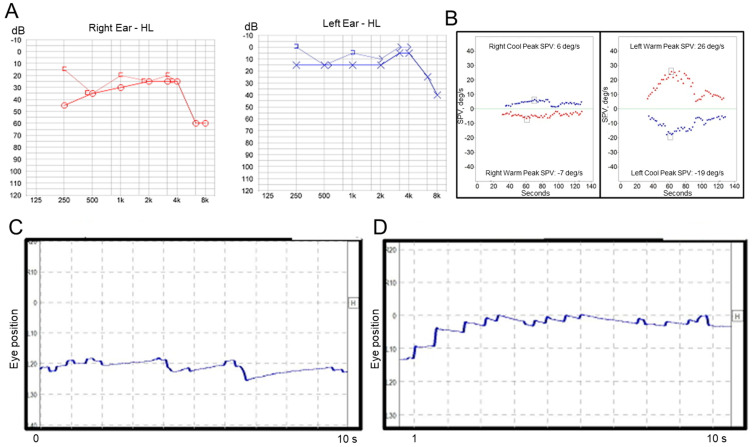
Results of laboratory tests in Case 2. (**A**) Pure tone audiometry shows mild sensorineural hearing loss on the right side. (**B**) A bithermal caloric test reveals canal paresis of 55% on the right side. Video-nystagmography demonstrates very weak left-beating nystagmus in the right head-roll position (**C**), and right-beating nystagmus in the left head-roll position (**D**).

A previously healthy 53-year-old woman first visited our clinic with symptoms of acute vertigo, which started 8 h prior to her visit to our clinic and lasted for 1 h, hearing loss, tinnitus, and aural fullness on the right side. She did not complain of headache and denied a past medical history of migraine. Otoscopic examination revealed a normal tympanic membrane, and neurological examination revealed no focal neurologic deficit. Pure tone audiometry revealed sensorineural hearing loss on the right side (Figure 2A), and a bithermal caloric test revealed canal paresis of 55% on the right side (Figure 2B). Video-nystagmography (Supplementary Video S2), which was performed 23 h after the vertigo subsided, demonstrated very weak, persistent apogeotropic positional direction-changing nystagmus in the right (SPV = 2°/s, Figure 2C) and left head-roll positions (SPV = 3°/s, Figure 2D). The patient showed no spontaneous nystagmus in a sitting position, and exhibited weakly right-beating nystagmus (SPV = 1°/s) in a bowing position and weakly left-beating nystagmus (SPV = 1°/s) in a leaning position. The patient was treated with systemic steroids and intratympanic steroid injections during the acute stage, and then maintained with betahistine and lifestyle modification. After treatment, the hearing loss improved. After two months, she visited the emergency department with a complaint of acute vertigo that had started 4 h prior and lasted for 2 h. The patient reported that she had experienced repetitive vertiginous attacks during the preceding two month-period. Examination using video Frenzel goggles, which was performed 5 h after the vertigo subsided, demonstrated weak right-beating nystagmus without direction change on positioning maneuvers. The patient was treated with systemic steroids, and a recurrent vertiginous attack occurred 19 months after the last treatment.

## 3. Discussion

Because the duration of each vertiginous attack is generally short in Meniere’s disease, most patients visit dizziness clinic during the interictal period. For this reason, only a limited number of studies have reported nystagmus findings during acute vertiginous attacks in Meniere’s disease, with discrepancies among the studies [3,4,5,6,7]. McClure et al., investigated nystagmus findings in eight patients with Meniere’s disease during a vertiginous attack, and reported, in 1981, that all of the patients showed an initial contralateral nystagmus (‘paralytic’ nystagmus) during the acute phase of the attack with reversal to an ipsilateral nystagmus (‘recovery’ nystagmus) as the acute symptoms subsided [6]. Meissner investigated nystagmus findings from 37 vertigo attacks in 20 patients with Meniere’s disease and reported, in 1981, that nystagmus toward the diseased ear (‘irritative’ nystagmus) without reversal was observed in 38% (14 of 37), nystagmus toward the diseased ear (‘irritative’ nystagmus) with reversal was observed in 38% (14 of 37), nystagmus toward the healthy ear (‘paralytic’ nystagmus) without reversal was observed in 8% (three of 37), and nystagmus toward the healthy ear (‘paralytic’ nystagmus) with reversal was observed in 16% (six of 37) of attacks [7]. He also observed that the direction of nystagmus during attacks may change inconsistently between the attacks in some patients [7]. Nishikawa and Nishikawa recorded nystagmus from before the vertiginous attack until its end in one patient with Meniere’s disease and reported, in 1986, that the nystagmus was tri-phasic, showing nystagmus beating toward the affected side before the start of the vertigo attack (‘irritative’ nystagmus), toward the non-affected side at the onset of the attack (‘paralytic’ nystagmus), and again toward the affected side in the middle and at the end of the attack (‘irritative’ nystagmus) [5]. Bance et al., observed the nystagmus at the very beginning of acute vertiginous attacks in two patients with Meniere’s disease and reported, in 1991, that both patients showed initial ipsilateral nystagmus (‘irritative’ nystagmus), which reversed to contralateral nystagmus (‘paralytic’ nystagmus) during the attacks [4]. Hirai et al., recorded nystagmus at the onset of a vertiginous attack in two patients with Meniere’s disease and reported, in 2017, that nystagmus was directed toward the affected side (‘irritative’ nystagmus) over the entire course of the vertiginous attacks in both of the patients [3]. Thus, the alteration of spontaneous nystagmus direction has been a characteristic vestibular finding during an acute vertiginous attack in Meniere’s disease, although the observed nystagmus is either ‘irritative’ or ‘paralytic’, according to the time point of nystagmus recording or individual difference. Although it is still controversial, the diffusion of endolymph potassium into the perilymph has been assumed to be the underlying mechanism of this direction-changing spontaneous nystagmus during attacks in Meniere’s disease [10,11].

On the other hand, Aschan and Stahle investigated the nystagmus findings in 21 patients with Meniere’s disease during attacks and reported, in 1957, that the nystagmus direction varied with the change in head position in five patients, of which two patients showed geotropic positional direction-changing nystagmus during head-roll positions [12]. Kim et al., investigated the nystagmus findings in 65 patients with Meniere’s disease during the interictal period and reported, in 2019, that positional direction-changing nystagmus was observed in 22% (14 of 65) of the patients, with geotropic and apogeotropic nystagmus in seven patients, respectively [13]. Secondary benign paroxysmal positional vertigo (BPPV) that was associated with Meniere’s disease has been reported in 8–10% of patients with Meniere’s disease, generally developing 2–5 years after the onset of Meniere’s disease [14,15,16,17,18]. In the present study, we demonstrated nystagmus findings within several hours after the end of acute vertiginous attack in two patients with unilateral definite Meniere’s disease. Our patients showed very weak, persistent positional direction-changing nystagmus, of which the direction was geotropic in Case 1 and apogeotropic in Case 2. This type of positional nystagmus has been reported in other inner ear disorders [19,20,21,22,23,24], and it could not be clearly explained by typical BPPV [25]. The change in chemical composition and/or electrolyte concentration in the inner ear fluid, although still unclear, may underlie the production of this characteristic nystagmus in these patients. Aschan et al., observed geotropic (early phase) and apogeotropic (late phase) positional nystagmus after alcohol intake, and proposed variations in the composition of endolymph as an explanation for positional nystagmus following alcohol ingestion [26]. Inflammation or intralabyrinthine traumatisms may cause time-dependent conditions, in which the cupula is lighter (light cupula) or heavier (heavy cupula) than the endolymph [27,28], and an analogous mechanism may explain the positional nystagmus that is observed immediately after vertigo attack in patients with Meniere’s disease. Another interesting finding was that the bithermal caloric test revealed canal paresis of 55% on the affected side in Case 2, and no canal paresis at the first visit and later canal paresis of 49% on the affected side in Case 1. It is not clear whether canal paresis is transient and secondary to a mechanical otoconia-related problem or to a vestibular nerve damage that is associated with duration and the number of attacks, as observed in BPPV [29]. Although the relationship between positional nystagmus and caloric response cannot be explicitly explained, further studies monitoring the caloric response with follow-up tests after several months in more patients with Meniere’s disease may clarify this relationship.

## 4. Conclusions

To the best of our knowledge, this study is the first to demonstrate the capture of positional direction-changing nystagmus immediately after an attack in Meniere’s disease. An analysis of nystagmus from the onset of a vertiginous attack through the interictal period in more patients with Meniere’s disease may contribute to advancing the research on the mechanism that underlies the symptoms of Meniere’s disease.

## Data Availability

Data in this study is available by request to the corresponding author.

## Supplementary Materials

The following are available online at https://www.mdpi.com/2039-4349/11/1/7/s1, Video S1. The patient with the left Meniere’s disease. Recording of eye movement, which was performed at 5 h after the vertigo subsided, demonstrated very weak, persistent geotropic positional direction-changing nystag-mus during a head-roll test. Video S2. The patient with the right Meniere’s disease. Recording of eye movement, which was performed at 23 h after the vertigo subsided, demonstrated very weak, persistent apogeotropic positional direction-changing nystagmus during a head-roll test.
