# Supplementary material for: Positional Nystagmus after Acute Vertiginous Attack in Meniere’s Disease
Source: Audiol Res. 2021 Feb 6;11(1):55–62. doi: 10.3390/audiolres11010007 (PMC7931113; doi:10.3390/audiolres11010007)
Supplement: Supplementary file 1 [file audiolres-11-00007-s001.zip › supplementary files/Certificate of English editing.pdf]

CERTIFICATE OF ENGLISH EDITING

This document certifies that the paper listed below has been edited to ensure that the language is clear and free of errors. The edit was performed by professional editors at Editage, a division of Cactus Communications.The intent of the author's message was not altered in any way during the editing process. The quality of the edit has been guaranteed, with the assumption that our suggested changes have been accepted and have not been further altered without the knowledge of our editors.

Title Of The Paper

Positional Nystagmus after Acute Vertiginous Attack in Meniere's Disease

Authors

Chang-Hee Kim

Job Code

RAOIT_6

editage by CACTUS

Signature

Vikas Narang

Vikas Narang,
Chief Operating Officer,
Editage

Date of Issue
January 28, 2021

Editage, a brand of Cactus Communications, offers professional English language editing and publication support services to authors engaged in over 500 areas of research. Through its community of experienced editors, which includes doctors, engineers, published scientists, and researchers with peer review experience, Editage has successfully helped authors get published in internationally reputed journals. Authors who work with Editage are guaranteed excellent language quality and timely delivery.

INFORMATION SECURITY MANAGEMENT SYSTEM DNV·GL ISO/IEC 27001

CACTUS.

Contact Editage

Worldwide
request@editage.com
+1(833)979-0061
www.editage.com

Japan
submissions@editage.com
+81 0120-50-2987
www.editage.jp

Korea
submit-korea@editage.com
02-3478-4396
www.editage.co.kr

China
fabiao@editage.cn
400-005-6055
www.editage.cn

Brazil
contato@editage.com
+5508000474773
www.editage.com.br

Taiwan
submitjobs@editage.com
02 2657 0306
www.editage.com.tw
