# Supplementary material for: Positional Nystagmus after Acute Vertiginous Attack in Meniere’s Disease
Source: Audiol Res. 2021 Feb 6;11(1):55–62. doi: 10.3390/audiolres11010007 (PMC7931113; doi:10.3390/audiolres11010007)
Supplement: Supplementary file 1 [file audiolres-11-00007-s001.zip › supplementary files/Fig. 2.pptx]

## Slide 1
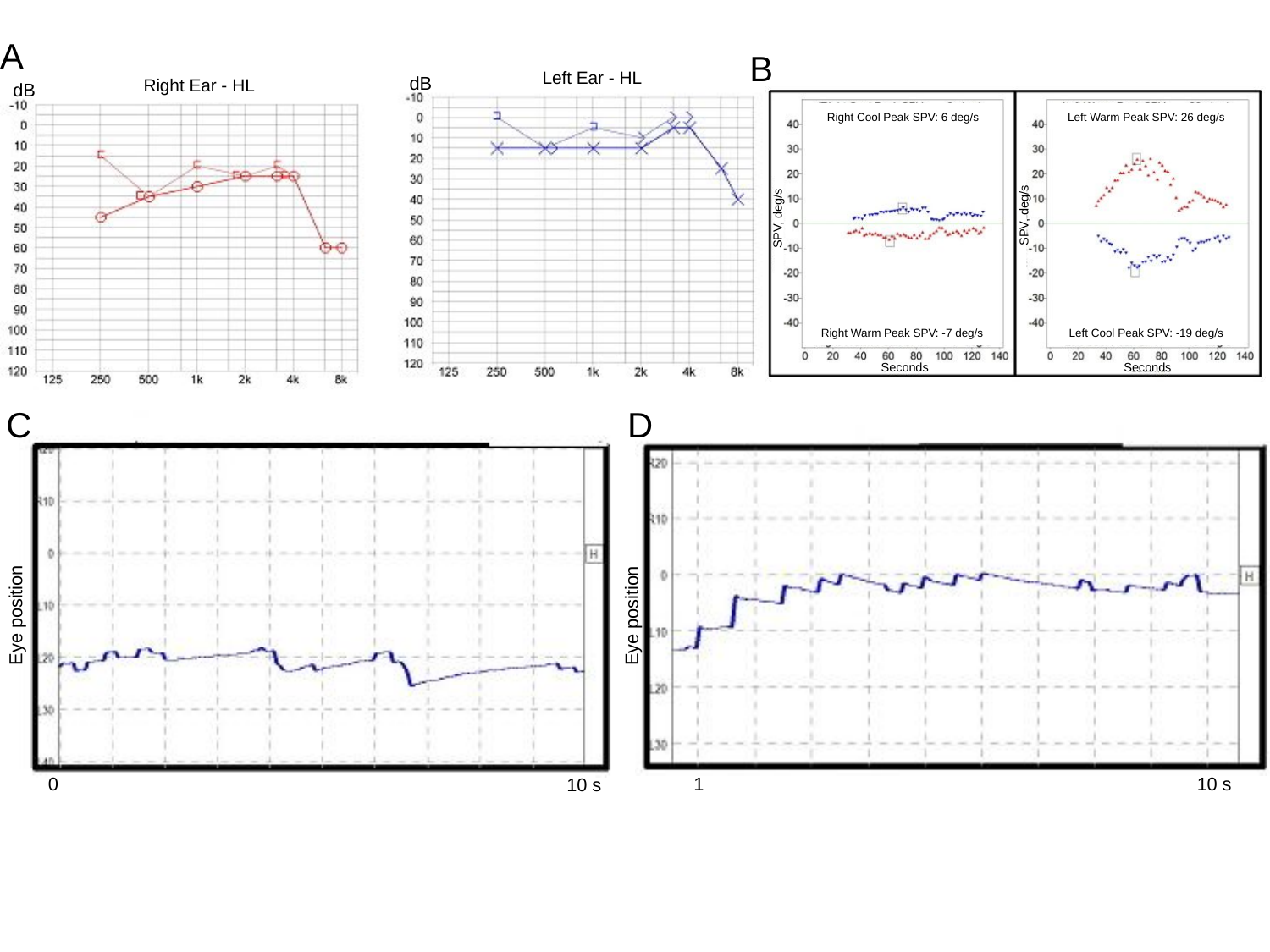

A
B
dB
dB
C
D
Eye position
Eye position
0
1
10 s
10 s
Left Ear - HL
Right Ear - HL
Right Cool Peak SPV: 6 deg/s
Left Warm Peak SPV: 26 deg/s
SPV, deg/s
SPV, deg/s
Right Warm Peak SPV: -7 deg/s
Left Cool Peak SPV: -19 deg/s
Seconds
Seconds
